# Genotype F of Echovirus 25 with multiple recombination pattern have been persistently and extensively circulating in Chinese mainland

**DOI:** 10.1038/s41598-024-53513-2

**Published:** 2024-02-08

**Authors:** Xiaoyi Wang, Jianping Cun, Shikang Li, Yong Shi, Yingying Liu, Haiyan Wei, Yong Zhang, Ruyi Cong, Tingting Yang, Wenhui Wang, Jinbo Xiao, Yang Song, Dongmei Yan, Qian Yang, Qiang Sun, Tianjiao Ji

**Affiliations:** 1 Medical School, Anhui University of Science and Technology, Huainan, 232001 China; 2 WHO WPRO Regional Polio Reference Laboratory, National Health Commission Key Laboratory for Biosafety, National Health Commission Key Laboratory for Medical Virology, National Institute for Viral Disease Control and Prevention, Chinese Center for Disease Control and Prevention, Beijing, 102206 China; 3 Yunnan Center for Disease Control and Prevention, Kunming, 650100 China; 4 Hunan Center for Disease Control and Prevention, Changsha, 410005 China; 5 Jiangxi Center for Disease Control and Prevention, Nanchang, 330006 China; 6 Hebei Center for Disease Control and Prevention, Shijiazhuang, 050000 China; 7 Henan Center for Disease Control and Prevention, Zhengzhou, 450000 China; 8 Shandong First Medical University (Shandong Academy of Medical Sciences) School of Public Health and Health Management, Jinan, 250117 China

**Keywords:** Viral epidemiology, Viral evolution, Retrovirus

## Abstract

Echovirus 25 (E25), a member of the Enterovirus B (EV-B) species, can cause aseptic meningitis (AM), viral meningitis (VM), and acute flaccid paralysis (AFP). However, systematic studies on the molecular epidemiology of E25, especially those concerning its evolution and recombination, are lacking. In this study, 18 strains of E25, isolated from seven provinces of China between 2009 and 2018, were collected based on the Chinese hand, foot, and mouth disease (HFMD) surveillance network, and 95 sequences downloaded from GenBank were also screened. Based on the phylogenetic analysis of 113 full-length VP1 sequences worldwide, globally occurring E25 strains were classified into 9 genotypes (A–I), and genotype F was the dominant genotype in the Chinese mainland. The average nucleotide substitution rate of E25 was 6.08 × 10^–3^ substitutions/site/year, and six important transmission routes were identified worldwide. Seventeen recombination patterns were determined, of which genotype F can be divided into 9 recombination patterns. A positive selector site was found in the capsid protein region of genotype F. Recombination analysis and pressure selection analysis for genotype F showed multiple recombination patterns and evolution characteristics, which may be responsible for it being the dominant genotype in the Chinese mainland. This study provides a theoretical basis for the subsequent prevention and control of E25.

## Introduction

Enteroviruses (EVs) belong to the genus Enterovirus in the family of small RNA viruses. Currently, there are 15 species of enteroviruses, EV-A–EV-L and RV-A–RV-C^1^. The whole enterovirus genome is approximately 7400 bps, consisting of a noncoding region (UTR) at both ends and a complete open reading frame (ORF). The ORF consists of a structural protein encoding the P1 region and two nonstructural proteins encoding the P2 and P3 regions^2^. P1 encodes four viral capsid proteins (VP1, VP2, VP3, and VP4), which are comparatively conserved, while P2 encodes three nonstructural proteins (2A, 2B, and 2C) and P3 encodes four nonstructural proteins (3A, 3B, 3C and 3D), which are highly variable and encode a variety of enterovirus proteases that play important roles in viral replication and host pathogenesis^3^.

Echovirus 25 (E25) is an important human pathogen belonging to the EV-B of enteroviruses and is associated with several clinical diseases, including aseptic meningitis (AM), viral meningitis (VM), acute flaccid paralysis (AFP) and hand, foot, and mouth disease (HFMD)^4^. The prototype strain of E25 (JV-4) was first discovered in 1957 in the USA^5^, and since the discovery of the prototype strain, E25 has been detected in several countries, including India^6^, China^7^, Australia^8^, the USA^9^, the United Kingdom^10^, France^11^, Italy^12^, Germany^13^, and Korea^14^. Although E25 has been detected in some national disease surveillance systems, sequences of E25 in GenBank are still relatively scarce, Moreover, there is currently no well-accepted classification system available for E25. In 2010, Chao Ling et al. classified two genotypes of E25 (genotypes A and B), based on nucleotide differences greater than 20%, with four E25 strains from Henan Province, China, and 13 E25 strains from GenBank^15^. In 2015, Li Hongjie et al. defined four lineages (A to D) based on one strain from Beijing, China, and 19 strains from GenBank^4^. Due to the small number of sequences included, the accuracy of the above study needs to be further investigated.

To date, studies on the genetic evolution, phylogenetic relationships, and spatiotemporal dynamics of E25 are still relatively scarce. In this study, we combined the 18 E25 isolates collected from the Chinese mainland with the full length of all E25 VP1 sequences retrieved from GenBank to construct a global gene sequence database of E25, thus providing a more systematic molecular epidemiological analysis of E25 on a global scale.

## Results

### Nine genotypes were reclassified based on the VP1 sequences

**Figure 1:**
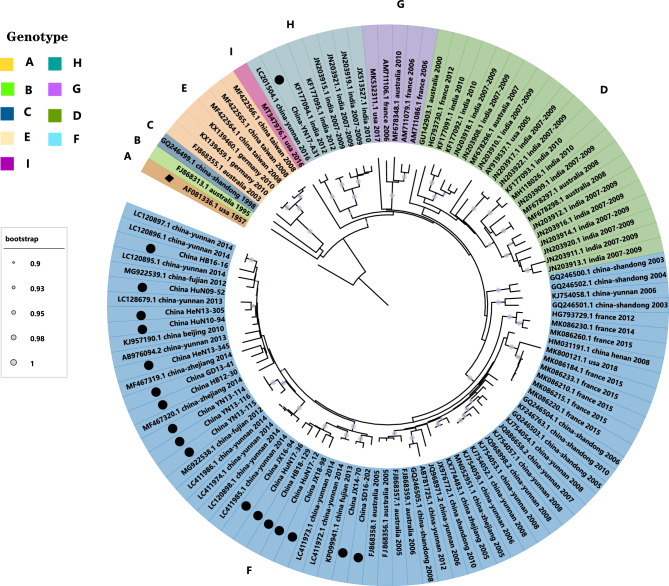
The maximum likelihood phylogenetic tree is based on the entire *VP1* genome of 113 E25 genome sequences. Filled circle: **⚫**E25 strains in this study. Filled rhombus: ◆E25 prototype strain.

According to the guidelines for genotype classification, 15–25% nucleotide differences between genotypes and less than 15% within genotypes^16^, 113 full-length VP1 sequences of E25 could be classified into nine genotypes (A–I) (Fig. 1). The nucleotide differences among the 9 genotypes ranged from 15.7 to 24.7%, and details about nucleotide and amino acid differences between the different genotypes are shown in Table 1.

**Table 1:** Information on 9 genotypes of E25 relying on full-length VP1 sequences.

Genotype	Number of sequences	Isolated country	Isolated year	Distance to external clusters		Intra-cluster distances	MCMC (BEAST)	
				Nucleotide	Amino acid	(Nucleotide)	Substitution rate (10-3) (95%HPD)	tMRCA (95% HPD)
ALL	113	USA, India, Australia, France, China, Germany	1957–2018	NA	NA	NA	6.08 (5.10–7.16)	1923 (1901–1944)
A	1	USA	1957	0.233–0.247	0.047–0.070	NA	NA	NA
B	1	Australia	1995	0.184–0.242	0.036–0.053	NA	NA	NA
C	1	China	1998	0.182–0.236	0.031–0.058	NA	NA	NA
D	22	USA, India, Australia, France	2000–2012	0.158–0.247	0.039–0.070	0.11	NA	NA)
E	6	China, Germany, Australia	2003–2010	0.182–0.241	0.031–0.055	0.03	NA	NA
F	68	China, France, Australia, USA	2003–2018	0.157–0.236	0.029–0.061	0.08	6.44 (5.53–7.34)	1993 (1989–1996)
G	5	Australia, France, USA	2006–2017	0.158–0.233	0.037–0.066	0.11	NA	NA
H	8	India, China	2008–2017	0.157–0.236	0.030–0.069	0.07	NA	NA
I	1	USA	2016	0.170–0.241	0.029–0.058	NA	NA	NA

The prototype strain (JV-4) was classified as genotype A, and the remaining eight genotypes were named sequentially in chronological order. Genotypes B, C, and I, all consisting of only one sequence, were isolated in Australia in 1995, China in 1998, and the USA in 2016, respectively. Genotype D included 22 sequences, and most of them (16/22) were isolated in India between 2010 and 2012. Similarly, genotype H was mainly composed of Indian strains (6/8) and two Chinese strains. Genotype E included 6 sequences from Australia, Germany, and Taiwan, China. The five sequences of genotype G were from Australia, France, and the USA. Genotype F included 68 sequences covering four countries, China, the USA, Australia, and France, of which 55 sequences were from China, and this genotype was also the main genotype prevalent in the Chinese mainland.

### Phylodynamic analysis of E25

**Figure 2:**
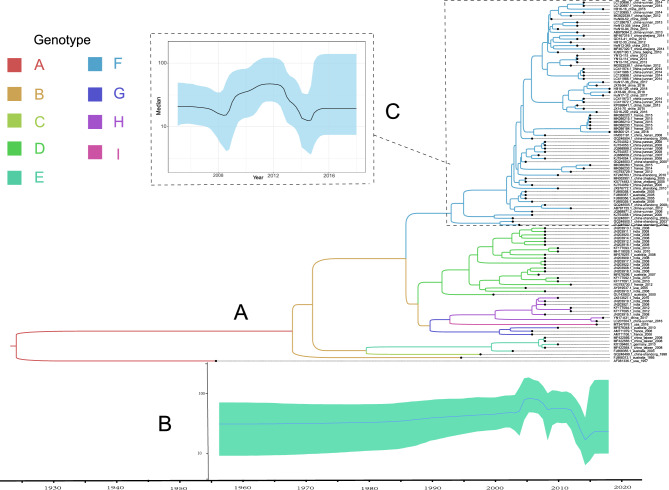
(**A**)The maximum clade credibility (MCC) phylogenetic tree was generated using the Markov chain Monte Carlo (MCMC) method based on 110 complete VP1 sequences of E25. The color of the branches represents the isolates of different genotypes. (**B**) Bayesian skyline plot of the whole E25 VP1 region sequence, reflecting the relative genetic diversity of E25 from 1957 to 2018. The X-axis is the time scale (year), and the Y-axis is the effective population size; the solid line is the estimated median, and the blue shadow is the 95% highest posterior density. (**C**) Bayesian skyline plot of theVP1 region sequence of E25 Genotype F.

Using BEAST software, 110 full-length E25 VP1 were analyzed for evolutionary origin. The results showed that the mean evolutionary rate of E25 was 6.08 × 10^–3^ substitutions/site/year (95% HPD: 5.10 × 10^–3^ to 7.16 × 10^–3^); the time of origin was 1923 (95% HPD: 1901 to 1944) (Table 1, Fig. 2A). Correspondingly, genotype F originated in approximately 1993 with an evolutionary rate of 6.44 × 10^–3^ substitutions/site/year (95% HPD:5.53 × 10^–3^ to 7.34 × 10^–3^) (Table 1). The Bayesian skyline plot shows that the E25 community size was stable and steady until 2004, with small increasing and decreasing fluctuations from 2004 to 2008; Then, there was a small increase after first starting to decline from 2012 to 2016; and a steady state after 2016 (Fig. 2B). The Genotype F showed a small fluctuation between 2008 and 2014 (Fig. 2C). However, the sequences of other genotypes are not suitable for analysis by BEAST alone because of the large errors caused by the small number of sequences.

### Six important global geographic transmission paths of E25

**Figure 3:**
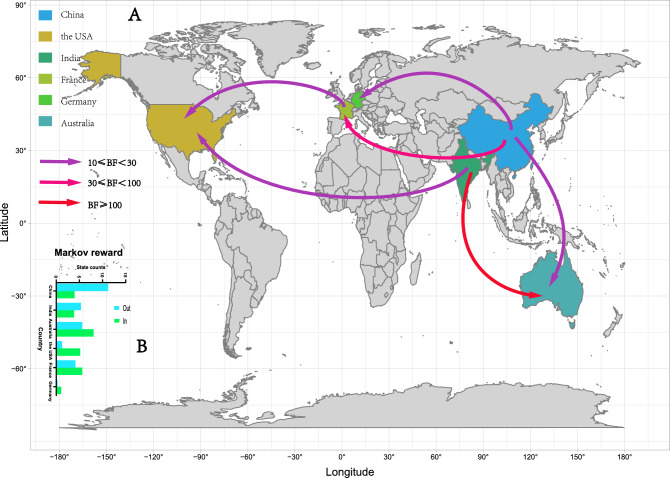
(**A**) The global spatial transmission route of E25. (**B**) The histogram of the average number of state transitions is based on the geographical location of six countries.

Based on the above 110 full-length VP1 sequences of E25, a sequence database that includes six countries (the USA, China, Australia, France, Germany, and India) was established to analyze the global spatiotemporal dynamics of E25. Based on the Bayes factor (BF) ≥ 10 and posterior probability (PP) ≥ 0.5, six significant transmission pathways were identified: China to Germany (BF = 23.88, PP = 0.85); China to France (BF = 37.23, PP = 0.90); China to Australia (BF = 21.56, PP = 0.83); India to the USA (BF = 12.24, PP = 0.74); India to Australia (BF = 400.25, PP = 0.99); and France to the USA (BF = 12.17, PP = 0.74) (Fig. 3A, Supplementary Table S3). The above pathways show that E25 primarily spreads from Asia to the rest of the world. In addition, the results of the Markov reward showed that China dominates the output of E25 worldwide with a Markov reward value of 11.32, which is much higher than other countries (Fig. 3B, Supplementary Table S4). However, the results are somewhat biased due to the limited the number of available world series for each country.

### Analysis of recombination patterns of E25

**Figure 4:**
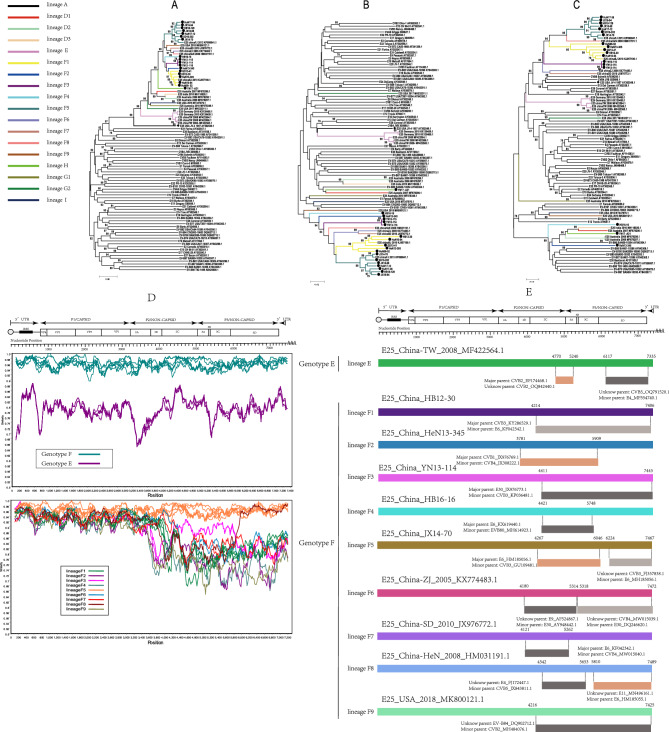
NJ trees based on P1, P2, and P3 regions of the prototype sequence of all Enteroviruses B in the GenBank database with 37 E25 strains. Filled rhombus: indicates E25 prototype strain (JV-4), filled circle: E25 strains in this study. Numbers on codes indicate the bootstrap support of the node (1000 bootstrap replicate percentage). (**A**) P1 coding sequences; (**B**) P2 coding sequences; (**C**) *P3* coding sequences. (**D**) Recombination breakpoints based on the whole genomes of different lineages as detected within genotypes. (**E**) Genomic mapping of representative strain recombination events predicted using RDP4 software for E25.

For a better understanding of the recombinant pattern of E25, a total of 37 sequences containing 7 genotypes (A, D–I) were used to construct phylogenetic trees based on the P1, P2, and P3 regions with the prototypes of other serotypes in the EVB group respectively. Among them, 18 sequences were obtained from this study and 19 were downloaded from GenBank (Fig. 4A–C). The results showed that all 37 strains of E25 clustered together in the P1 region and branched in the P2 and P3 regions. According to the position of sequences with varying genotypes on the phylogenetic tree in the P2 and P3 regions, we define that, if the nucleotide differences among the different linages were more than 15% it could be recognized as a valid recombination lineage. We defined 18 lineages (including one prototype lineage) to facilitate the analysis of their recombination patterns (Fig. 4A–C, Supplementary Table S5), and Genotype F could be divided into nine lineages (lineage F1–F9), while Genotype E had one lineage (lineage E). Analysis using Simplot software revealed significant differences in nucleotide similarity between Genotype E and Genotype F, with the nine lineages of Genotype F showing large differences in the P2 and P3 regions (Fig. 4D). This is also consistent with the results of the phylogenetic trees constructed by P2 and P3 and also shows that the recombination pattern differs between different lineages. According to the differences among these lineages, 17 recombination patterns were identified, of which Genotype F can be divided into 9 recombination patterns. For further validation, we randomly selected one strain from each of these 17 lineages as the reference sequence and used RDP4 software for recombination analysis, which showed that the breakpoint position information of each of the 17 reference sequences was different, and in addition, the serotypes of the prevalent strains that recombined with the seventeen reference strains also differed significantly (Fig. 4E, Supplementary Table S6). This also confirms that the reorganization patterns of the 17 lineages classified are indeed different.

### A positive selection site was detected in genotype F

**Figure 5:**
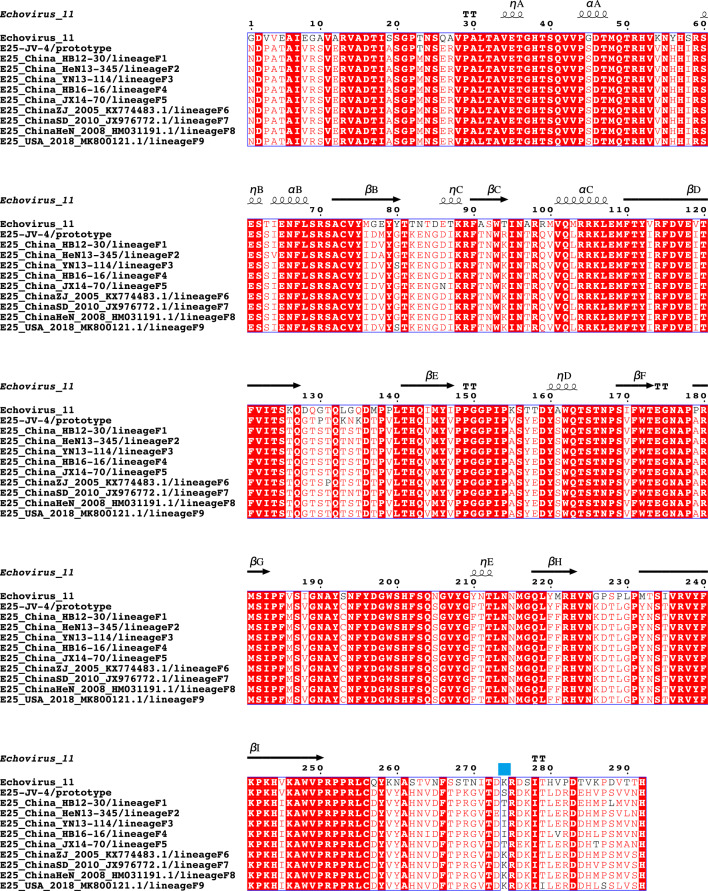
Positive amino acid selector sites in the VP1 region of E25. Positive selector sites are located at the blue square markers.

Since the P1 region sequences of other genotypes were rare or absent (Supplementary Table S6), we selected Genotype F, which had a relatively large number of sequences, for analysis. By analyzing the selection pressure of global Genotype F globally, we found that the average ratio of nonsynonymous to synonymous amino acid substitutions (dN/dS) in the P1 region was 0.207. There was a positive selection site at amino acid position 274 in the VP1 region, which was identified by both the MEME and SCLA models. Moreover, the amino acids at this site also differed among the reference strains on the 9 different lineages of Genotype F (Fig. 5).

## Discussion

As one of the earliest enteroviruses discovered in the world, E25 is still identified by enterovirus surveillance systems in some countries, and there is a risk of it causing outbreaks in the population. In contrast to its risk, systematic research on the molecular epidemiology of E25 is lacking, especially for those related to evolution and recombination. Therefore, we performed a global-wide analysis using 113 complete VP1 sequences of E25 from 1957 to 2018, together with 18 whole-genome sequences obtained in this study to provide a comprehensive molecular characterization and recombination analysis of E25.

Genotyping analysis can provide a theoretical basis for studying genetic characteristics and molecular evolution principles. In this study, we classified the global E25 full-length VP1sequences into nine genotypes. Compared with previous studies^4,15^, we established a much larger dataset including more sequences with more countries and disease types. In addition, according to published genotyping criteria of other thoroughly studied enteroviruses, our analysis of the genotypes among different regions by more rigorous classification criteria makes the results more reliable. Genotype F is the predominant genotype in the Chinese mainland and has been spreading in the Chinese mainland for 20 years. We also found that different evolutionary branches formed in the ML tree, which indicates that Genotype F continued to mutate and evolve during these years, and has the potential risk of causing outbreaks in the future.

BEAST analysis showed that the evolutionary rate of E25 is close to the evolutionary rate of some other enteroviruses^17–19^. Moreover, the evolutionary rate of genotype F was approximately the same as the total average rate of E25, which indicates that other genotypes of E25 have not undergone large-scale mutation and evolution, although the rate and ancestor cannot be estimated due to the small size of sequences in this study. From the community dynamic distribution map, we found that the E25 community size experienced a small change from 2004 to 2008, but did not cause large-scale disease outbreaks based on a literature search. This partly reflects the fact that E25 outbreaks and epidemics are easily overlooked and that the disease burden may be underestimated. Therefore, it is particularly important to strengthen the surveillance and monitoring of enteroviruses, especially the screening and detection of other enteroviruses, in the external environment and the population. Six important dissemination paths of E25 were found worldwide, and three of them involved export from China; they were strongly supported by the BF value and were consistent with the Markov reward results. Although there is a certain selection bias due to the large number of Chinese sequences, this result also reflects that with the frequent development of world trade, the cross-transmission of infectious diseases, especially the transmission of different viruses or different genotypes of viruses, cannot be ignored.

In addition, a positive selector site was found in the capsid protein region of genotype F by selective pressure analysis in E25 capsid protein. The VP1 structural protein contains some important antigenic sites and changes in amino acids in this region may affect viral virulence^20,21^, we speculated that changes in this site may increase the ability of the virus to invade the host. Therefore, the pathogenicity and transmission of the Genotype F virus in the population are improved, but further animal experiments are needed to verify this.

Although we have collected the sequences of E25 in GenBank as much as possible, after our screening and verification, we found that there are few effective sequences that can be used. Among these, sequences that collected from China have the most and complete information, which leads to a certain bias in sequence selection. Similarly, as most of the complete genome sequences in GenBank (10/19) lack important information including disease type and isolation origins, the recombination analysis in this study only compared the different recombination patterns among different genotypes. Meanwhile, although we attempted to conduct a geographic spreads analysis covering more countries by basing on partial VP1 sequences, we found that this would sacrifice more important nucleotide sites (requiring a reduction of more than 500 bp). Therefore, this study only provides a systematic bioinformatics analysis based on full-length of VP1, which also causes a certain limitation.

In summary, we identified E25 genotyping criteria and successfully classified 9 genotypes according to these criteria in this study. We analyzed the evolutionary origin and spatial transmission of E25 worldwide, and different genotypes showed different epidemic characteristics. Recombination analysis and pressure selection analysis of genotype F showed that there are more multiple recombination patterns in the P2 and P3 regions and a positive selector site in the capsid protein region. These factors may be responsible for Genotype F being the dominant genotype in the Chinese mainland. This study provides a theoretical basis for the subsequent prevention and control of E25.

## Methods

### Virus isolation

The 18 clinical samples in this study were based on the Chinese HFMD surveillance network and were isolated from seven provinces in the Chinese mainland between 2009 and 2018 (Supplementary Table S1). Among these, one was isolated from a fatal case of HFMD, three were isolated from severe cases of HFMD, and the rest were collected from mild cases. Specimen processing and virus isolation were performed according to the requirements of the Chinese "Guidelines for the Prevention and Control of Hand, Foot and Mouth Disease (2009 edition)" (https://www.gov.cn/gzdt/2009-06/04/content_1332078.htm. Accessed 15 Apr 2023). Human rhabdomyosarcoma (RD) cells were used for virus isolation and amplification. RD cells were provided by the American Center for Disease Control and Prevention. Cytopathic effects (CPE) were observed within one week after cell infection, and samples were collected and frozen (− 80 °C) for long-term storage.

### E25 whole-genome sequencing

Viral RNA was extracted using a QIAamp viral RNA Mini Kit (Qiagen, Valencia, CA, the USA), following the protocol provided by the manufacturer. Then, we amplified the whole E25 genome by reverse transcription-polymerase chain reaction (RT-PCR) using a PrimeScript One-Step RT-PCR Kit Ver.2 (TaKaRa, Shiga, China) and 18 E25 strains using primers of our design (Supplementary Table S2). PCR products were purified using the QIAquick PCR Purification Kit (Qiagen, Hilden, Germany) and then amplicons were sequenced in both directions using the ABI 3130 Genetic Analyzer (Applied Biosystems, Foster City, CA, the USA). Primers design for amplifying the full length of E25 were designed using NCBI, and primers for 5' UTR and 3' UTR amplification were obtained from other sources^22^. Fragment sequences were edited and spliced using Sequence 5.4.5 software (Gen Codes, Ann Arbor, MI, the USA). Sequences are stored in the China National Microbiology Data Center (NMDC) with accession numbers NMDCN0001DRD–NMDCN0001DRU (https://nmdc.cn/resource/genomics/sequence).

### Dataset establishment

Up to now, a total of 113 full-length VP1 sequences, of which 95 were from GenBank and 18 were from this study, were used for phylogenetic analysis after screening and excluding some sequences with obvious sequencing errors or high homology (Table S1). These sequences originate from six countries and span the years 1957 to 2018, with 61 Chinese strains isolated from 1998 to 2018. Sequence alignment was performed using the ClustalW tool (Sudhir Kumar, Arizona State University, Tempe, Arizona, the USA) in MEGA (version 7.0), and the phylogenetic evolutionary tree was constructed using the maximum likelihood method (ML) with bootstrap set to 1000 replicates. The best nucleotide substitution model was selected using jModeltest (version 2.1), and model selection (maximum composite likelihood) was performed^23^.

### Phylogenetic and spatiotemporal dynamics analysis of E25

To understand the origin time, evolutionary rate, and spatial transmission path of E25. A total of 110 full-length VP1 (including 17 cutting from whole genome) sequences, which were all within the valid interval according to the TemEst test, were selected (Supplementary Table S1). We used the Bayesian Markov-Monte Carlo chain (MCMC) method in BEAST (version 1.10.4) to estimate the evolutionary rate and time of origin of E25 with a relaxed molecular clock^24^. The Tracer program (version 1.7.1) was used to assess the convergence of the chain and the appropriate quality control parameters for the effective sample size (ESS > 200)^25^. Maximum branch confidence (MCC) trees were constructed using TreeAnnotator (version 1.10.4) and the top 10% of sampled trees were removed using the aging option, and the resulting trees were visualized using FigTree (version 1.4.3)^26^. The spreaD3 package (version 0.9.7) was used for the visual analysis of spatial transmission paths^27^. The generated log files were used to calculate the Bayes factor (BF), and the worldwide migration paths of E25 were summarized based on the metrics of BF ≥ 10, and posterior probability (PP) ≥ 0.5.

### Recombinant analysis of E25

As of 7 Feb 2023, GenBank has released 19 genome sequences of E25. Together with the 18 sequences comprehending in this study, a total of 37 whole genome sequences of E25 have been collected to conduct the recombinant analysis. The phylogenetic evolutionary trees of the P1, P2, and P3 regions were constructed with MEGA (version 7.0) using the Kimura2-parameter model with a bootstrap set to 1000 replicates, based on 18 whole-genome sequences of E25 from this study, 19 whole-genome sequences of E25 from GenBank, and other EV-B prototype strains. The full-length sequences with similarity higher than 85% in GenBank were obtained as potential recombinant parents by BLASTing the coding regions of each segment of the selected reference sequences separately. The selected full-length sequences were analyzed for recombination using the seven methods, RDP, GENECONV, 3Seq, Chimaera, SiScan, MaxChi, and LARD of RDP4 (version 4.101). Recombination events identified by at least four of these recombination methods and satisfying a p value less than 0.05 were recognized^28^. Simplot (version 3.5.1) software was used to visualize the similarity between sequences, Simplot correlation parameters were set as a window: 200 bp; step: 20 bp; T/t: 2.0; and nucleotide substitution model: Kimura (2-parameter)^29^.

### Selective pressure analysis in E25 capsid protein

The ratio of nonsynonymous to synonymous amino acid substitutions (dN/dS) is a useful indicator of the strength of natural selection acting on protein-coding genes. Calculation of dN/dS ratios for correlated sequence datasets using the mixed-effects model of evolution (MEME) and single-likelihood ancestor counting (SLAC) was performed on the Datamonkey website^30,31^ (http://www.Datamonkey.org, Accessed 5 March 2023. The SWISS-MODEL online tool was used to construct a structural model of the E25 coat protein^27^ (https://swissmodel.expasy.org, Accessed 20 March 2023), and the VP1 protein of E11 (PDB:8B8R) was chosen for this study. Based on this model, multiple sequence comparison results containing secondary structures were generated using the ESPript (v3.0) online tool (https://espript.ibcp.fr/ESPript/ESPript/index.php, Accessed 24 March 2023) for visualization and analysis^32^.

### Ethics statement

This study did not involve human participants or human experimentation. Only specimen (stool samples, throat swab samples) collected from HFMD patients for public health purposes at the urging of the Ministry of Health, P. R. of China. Written informed consent for the use of their clinical samples was obtained from the parents of the children whose samples were analyzed. This study was approved by the second session of the Ethics Review Committee of the National Institute for Viral Disease Control and Prevention (NIVDC), Chinese Center for Disease Control and Prevention, all experimental protocols were approved by NIVDC, and the methods were carried out in accordance with the approved guidelines.

### Supplementary Information

Supplementary Tables.

## Data Availability

Eighteen Sequences of E25 are stored in China National Microbiology Data Center (NMDC) with accession numbers NMDCN0001DRD–NMDCN0001DRU (https://nmdc.cn/resource/genomics/sequence).
