# Supplementary material for: Genotype F of Echovirus 25 with multiple recombination pattern have been persistently and extensively circulating in Chinese mainland
Source: Sci Rep. 2024 Feb 8;14:3212. doi: 10.1038/s41598-024-53513-2 (PMC10853551; doi:10.1038/s41598-024-53513-2)
Supplement: Supplementary file 1 — Supplementary Tables. [file 41598_2024_53513_MOESM1_ESM.docx]

Genotype F of Echovirus 25 with multiple recombination pattern have been persistently and extensively circulating in Chinese mainland

Xiaoyi Wang^1^, Jianping Cun^3^, Shikang Li^4^, Yong Shi^5^, Yingying Liu^6^, Haiyan Wei^7^, Yong Zhang^2^, Ruyi Cong^8^, Tingting Yang^8^, Wenhui Wang^8^, Jinbo Xiao^2^, Yang Song^2^, Dongmei Yan ^2^, Qian Yang^2^, Qiang Sun^2^, Tianjiao Ji^2*^

^1^ Medical School, Anhui University of Science and Technology, Huainan 232001, China;

^2^ WHO WPRO Regional Polio Reference Laboratory, National Health Commission Key Laboratory for Biosafety, National Health Commission Key Laboratory for Medical Virology, National Institute for Viral Disease Control and Prevention, Chinese Center for Disease Control and Prevention, Beijing 102206, China;

^3^ Yunnan Center for Disease Control and Prevention, Kunming 650100, China;

^4^ Hunan Center for Disease Control and Prevention, Changsha 410005, China;

^5^ Jiangxi Center for Disease Control and Prevention, Nanchang 330006, China;

^6^ Hebei Center for Disease Control and Prevention, Shijiazhuang 050000, China;

^7^ Henan Center for Disease Control and Prevention, Zhengzhou 450000, China;

^8^ Shandong First Medical University (Shandong Academy of Medical Sciences) School of Public Health and Health Management, Jinan 250117，China;

^*^ Correspondence: Tianjiao Ji, jitj@ivdc.chinacdc.cn

**Table S1.** 113 isolates used for analysis of E25

| **Genotype** | **Strain Name** | **Specimen Source** | **Disease type** | **GenBank Accession** | **Isolation Country** | **Isolation Year** | **Source** |
| --- | --- | --- | --- | --- | --- | --- | --- |
| A | JV-4 | / | / | AF081336.1 | the USA | 1957 | GenBank |
| B | 95.115.0262 | / | / | FJ868313.1 | Australia | 1995 | GenBank |
| C | 98279/SD/CHN/1998/E25 | Stool | / | GQ246499.1 | China | 1998 | GenBank |
| D | 10548b | / | / | AY919537.1 | the USA | 2005 | GenBank |
| D | CF233036/TH_3.76_FRA12 | Throat swab | / | HG793730.1 | France | 2012 | GenBank |
| D | 00.109.2915 | / | / | GU142903.1 | Australia | 2000 | GenBank |
| D | NSW-V07-2007-ECHO25 | Stool | / | MF678296.1 | Australia | 2007 | GenBank |
| D | NSW-V08-2008-ECHO25 | Stool | / | MF678297.1 | Australia | 2008 | GenBank |
| D* | NSW-V09-2008-ECHO25 | Stool |  | MF678298.1 | Australia | 2008 | GenBank |
| D | N-438 | / | / | JN203918.1 | India | 2008 | GenBank |
| D | N-706 | / | / | JN203920.1 | India | 2008 | GenBank |
| D | N-418 | / | / | JN203917.1 | India | 2008 | GenBank |
| D | N-356 | / | / | JN203914.1 | India | 2008 | GenBank |
| D | N-315 | / | / | JN203912.1 | India | 2008 | GenBank |
| D | N-326 | / | / | JN203913.1 | India | 2008 | GenBank |
| D | N-273 | / | / | JN203911.1 | India | 2008 | GenBank |
| D | N-171 | / | / | JN203910.1 | India | 2008 | GenBank |
| D | N-404 | / | / | JN203916.1 | India | 2008 | GenBank |
| D | N-862 | / | / | JN203922.1 | India | 2008 | GenBank |
| D | N-146 | / | / | JN203908.1 | India | 2008 | GenBank |
| D | N-156 | / | / | JN203909.1 | India | 2008 | GenBank |
| D | A36-43PD | Stool | / | KF177091.1 | India | 2010 | GenBank |
| D | A79-33PI | Stool | / | KF177093.1 | India | 2010 | GenBank |
| D | A36-44PD | Stool | / | KF177092.1 | India | 2010 | GenBank |
| D | 10-4339-2 | / | / | MH118026.1 | India | 2010 | GenBank |
| E | 03.097.1251 | / | / | FJ868355.1 | Australia | 2003 | GenBank |
| E | 61242-1563 | / | / | MF422565.1 | China | 2008 | GenBank |
| E | 61241-70868 | / | / | MF422564.1 | China | 2008 | GenBank |
| E | 61243-1564 | / | / | MF422566.1 | China | 2008 | GenBank |
| E* | Jena/AN1380/10 | Stool | AM | KX139459.1 | Germany | 2010 | GenBank |
| E | Jena/VI10308/10 | Stool | AM | KX139460.1 | Germany | 2010 | GenBank |
| F | 03356/SD/CHN/2003/E25 | Stool | / | GQ246501.1 | China | 2003 | GenBank |
| F | 03312/SD/CHN/2003/E25 | Stool | AM | GQ246500.1 | China | 2003 | GenBank |
| F | 04364/SD/CHN/2004/E25 | Stool | AM | GQ246502.1 | China | 2004 | GenBank |
| F | ZJ/ZuJ/F89/05/E25 | / | AM | MN052951.1 | China | 2005 | GenBank |
| F | E25/ZE-wly/Zhejiang/CHN/2005 | / | / | KX774483.1 | China | 2005 | GenBank |
| F | 05357/SD/CHN/2005/E25 | Stool | AM | GQ246503.1 | China | 2005 | GenBank |
| F | 169-YN-2006AFP | Stool | AFP | JQ968971.2 | China | 2006 | GenBank |
| F | 06402/SD/CHN/2006/E25 | Stool | AM | GQ246504.1 | China | 2006 | GenBank |
| F | 210/YN/06AFP/E25 | Stool | AFP | KJ754058.1 | China | 2006 | GenBank |
| F | 211/YN/06AFP/E25 | Stool | AFP | KJ754059.1 | China | 2006 | GenBank |
| F | 156-YN-2007AFP | Stool | AFP | JQ886658.2 | China | 2007 | GenBank |
| F | 184/YN/08AFP/E25 | Stool | AFP | KJ754057.1 | China | 2008 | GenBank |
| F | 34/YN/08AFP/E25 | Stool | AFP | KJ754052.1 | China | 2008 | GenBank |
| F | 154/YN/08AFP/E25 | Stool | AFP | KJ754053.1 | China | 2008 | GenBank |
| F | 33-YN-2008AFP | / | AFP | JQ968998.2 | China | 2008 | GenBank |
| F | 156/YN/08AFP/E25 | Stool | AFP | KJ754054.1 | China | 2008 | GenBank |
| F | 08094C5/SD/CHN/2008/E25 | Stool | AM | GQ246505.1 | China | 2008 | GenBank |
| F | HN-2 | / | VM | HM031191.1 | China | 2008 | GenBank |
| F | HuN09-52 | Stool | HFMD | / | China | 2009 | this study |
| F | E25/2010/CHN/BJ | / | HFMD/encephalitis | KJ957190.1 | China | 2010 | GenBank |
| F | 022/JN/CHN/AM/07/E25 | Cerebrospinal fluid | AM | KF246763.1 | China | 2010 | GenBank |
| F | HuN10-94 | Stool | HFMD | / | China | 2010 | this study |
| F | E25SD2010CHN | / | HFMD | JX976772.1 | China | 2010 | GenBank |
| F | HB12-30 | Rectal swab | HFMD | / | China | 2012 | this study |
| F | Echo25/FJZZ210/CHN/2012 | / | HFMD | MG922539.1 | China | 2012 | GenBank |
| F | 13-YN-2012 | Stool | / | AB781725.1 | China | 2012 | GenBank |
| F | Echo25/FJPT354/CHN/2012 | / | HFMD | MG922538.1 | China | 2012 | GenBank |
| F | 142B-YN-CHN-2013 | / | Viral Meningitis | LC128679.1 | China | 2013 | GenBank |
| F | 298-Sewage-YN-CHN-2013 | Sewage | / | AB976094.2 | China | 2013 | GenBank |
| F | HeN13-305 | Stool | HFMD | / | China | 2013 | this study |
| F | YN13-115 | Stool | HFMD | / | China | 2013 | this study |
| F | HeN13-345 | Stool | HFMD | / | China | 2013 | this study |
| F | YN13-114 | Stool | HFMD | / | China | 2013 | this study |
| F | GD13-41 | Stool | HFMD | / | China | 2013 | this study |
| F | YN13-116 | Stool | HFMD | / | China | 2013 | this study |
| F | XM0297 | Throat swab | / | KP099941.1 | China | 2013 | GenBank |
| F | 154-YN-CHN-2014JK | / | / | LC120897.1 | China | 2014 | GenBank |
| F | N026-YN-CHN-2014 | / | / | LC411986.1 | China | 2014 | GenBank |
| F | G010-YN-CHN-2014 | / | / | LC411973.1 | China | 2014 | GenBank |
| F | HY/26/14/E25 | / | AM | MF467319.1 | China | 2014 | GenBank |
| F | 291-YN-CHN-2014JK | / | / | LC120898.1 | China | 2014 | GenBank |
| F | JX14-70 | Rectal swab | HFMD | / | China | 2014 | this study |
| F | HY/8/14/E25 | / | AM | MF467320.1 | China | 2014 | GenBank |
| F | G042-YN-CHN-2014 | / | / | LC411974.1 | China | 2014 | GenBank |
| F | 151-YN-CHN-2014JK | / | / | LC120896.1 | China | 2014 | GenBank |
| F | 59-YN-CHN-2014JK | / | / | LC120895.1 | China | 2014 | GenBank |
| F | G008-YN-CHN-2014 | / | / | LC411972.1 | China | 2014 | GenBank |
| F | N008-YN-CHN-2014 | / | / | LC411985.1 | China | 2014 | GenBank |
| F | SD16-202 | Stool | HFMD | / | China | 2016 | this study |
| F | JX16-94 | Rectal swab | HFMD | / | China | 2016 | this study |
| F | HB18-129 | Nasopharyngeal | HFMD | / | China | 2018 | this study |
| F | HB16-16 | Rectal swab | HFMD | / | China | 2016 | this study |
| F | HuN17-12 | Stool | HFMD | / | China | 2017 | this study |
| F | HuN17-36 | Rectal swab | HFMD | / | China | 2017 | this study |
| F | JX18-98 | Rectal swab | HFMD | / | China | 2018 | this study |
| F | 05.109.3930 | / | / | FJ868356.1 | Australia | 2005 | GenBank |
| F | 05.210.0488 | / | / | FJ868358.1 | Australia | 2005 | GenBank |
| F | 05.136.4150 | / | / | FJ868357.1 | Australia | 2005 | GenBank |
| F | 06.048.1621 | / | / | FJ868359.1 | Australia | 2006 | GenBank |
| F | CF188073/PL_4.28_FRA12 | Plasma | Meningitis | HG793729.1 | France | 2012 | GenBank |
| F | CF314308113_FRA14-11-04_E25 | / | Neonate infection | MK086230.1 | France | 2014 | GenBank |
| F | CF315202070_FRA15-07-18_E25 | / | Neonate infection | MK086233.1 | France | 2015 | GenBank |
| F | CF315194071_FRA15-07-12_E25 | / | Fever | MK086260.1 | France | 2015 | GenBank |
| F | CF315196002_FRA15-07-14_E25 | / | Meningitis | MK086215.1 | France | 2015 | GenBank |
| F | CF315177020_FRA15-06-25_E25 | / | Meningitis | MK086184.1 | France | 2015 | GenBank |
| F | CF315177084_FRA15-06-26_E25 | / | Neonate infection | MK086220.1 | France | 2015 | GenBank |
| F | CF315197075_FRA15-07-14_E25 | / | Diarrhea | MK086210.1 | France | 2015 | GenBank |
| F | USA/2018-23126 | Cerebrospinal fluid | Acute Flaccid Myelitis | MK800121.1 | the USA | 2018 | GenBank |
| G | CF314038-06 | Cerebrospinal fluid | Meningitis | AM711106.1 | France | 2006 | GenBank |
| G | CF151101-06 | Cerebrospinal fluid | Meningitis | AM711079.1 | France | 2006 | GenBank |
| G* | CF205083-06 | Cerebrospinal fluid | Meningitis | AM711086.1 | France | 2006 | GenBank |
| G | NSW-V58-2010-ECHO25 | Stool | / | MF678348.1 | Australia | 2010 | GenBank |
| G | USA/CA/RGDS-2017-1010 | Cerebrospinal fluid | Hyperbilirubinemia | MK532311.1 | the USA | 2017 | GenBank |
| H | N-491 | / | / | JN203919.1 | India | 2008 | GenBank |
| H | N-401 | / | / | JN203915.1 | India | 2008 | GenBank |
| H | N-831 | / | / | JN203921.1 | India | 2008 | GenBank |
| H | A253D | / | / | JX513527.1 | India | 2010 | GenBank |
| H | A120-14a | Stool | / | KF177094.1 | India | 2012 | GenBank |
| H | A120-15a | Stool | / | KF177095.1 | India | 2012 | GenBank |
| H | 131R-YN-CHN-2016HC | Stool | / | LC201504.1 | China | 2016 | GenBank |
| H | YN17-A31 | Stool | HFMD | / | China | 2017 | this study |
| I | USA/2016-19521 | / | Acute flaccid myelitis | MT347976.1 | the USA | 2016 | GenBank |

Note: Sequences marked with * in Genotype were not used for Bayesian origin evolution and spatiotemporal dynamics analysis

**Table S2.** The primers designed in this study for amplifying and sequencing the whole genome sequence of E25

| Primer | Position | Sequence (5’~3’) | Orientation |
| --- | --- | --- | --- |
| 001S48[20] | 1~20 | GGGGACAAGTTTGTACAAAAAAGCAGGCTTTAAAACAGCTCTGGGGTT | Forward |
| 875R | 858~875 | CTTGCCTGTTTGCTGAAT | Reverse |
| 756F | 756-773 | GTGTCAACGCAGAAAACC | Forward |
| 1710R | 1692~1710 | CGTTGTATTCGGCACACAT | Reverse |
| 1500F | 1500~1519 | ATCTACCCTCATCAGTGGAT | Forward |
| 2430R | 2411~2430 | GTGATATGAATGGCGTGTCC | Reverse |
| 2269F | 2251~2269 | AGACAAACTACCGCTTCGT | Forward |
| 3052R | 3034~3052 | GAAATGTGACCAGCCATCAT | Reverse |
| 2983F | 2983~3001 | CTCGCATGTCAATACCCTT | Forward |
| 3852R | 3833~3852 | GGTTGACTTGTTCGCAGATT | Reverse |
| 3742F | 3742~3761 | TGCTGTGGTTGGAGGATGAT | Forward |
| 4661R | 4642~4661 | AACACTCGACACCATCTGAC | Reverse |
| 4533F | 4533~4553 | TATTCTCTACCACCAGACCCT | Forward |
| 5463R | 5444~5463 | ACTCACCATACTCTGTCTTC | Reverse |
| 5173F | 5173~5192 | AAGAGAATGGATGGCTCGTT | Forward |
| 6176R | 6157~6176 | ATCCACGGCTTCCATCATGT | Reverse |
| 5850F | 5850~5869 | ACTGGTAAGGTGTTGGGGAT | Forward |
| 6667R | 6649~6667 | GGGCTCAAACTGGCATCAT | Reverse |
| 6471F | 6471~6490 | GCATCCAGCCTGAACGACTC | Forward |
| 7419R | 7400~7419 | GCGGAGAATTTACCCCTA | Reverse |
| 7188F | 7188~7207 | TCTTTGTGCTTGTTGGCCTG | Forward |
| 7500A[20] | 7429~7453 | GGGGACCACTTTGTACAAGAAAGCTGGG(T)_24_ | Reverse |

**Table S3.** Migration paths based on the value of Bayes factor and posterior probability

| FROM | TO | BAYES_FACTOR | POSTERIOR PROBABILITY |
| --- | --- | --- | --- |
| Australia | China | 3.160844784 | 0.425397178 |
| Australia | France | 1.972135534 | 0.315964893 |
| Australia | Germany | 1.004965788 | 0.190534385 |
| Australia | India | 1.346422065 | 0.239751139 |
| Australia | the USA | 1.925849073 | 0.31085435 |
| China | France | 37.23127115 | 0.897122542 |
| China | Germany | 23.8841389 | 0.848350183 |
| China | India | 3.012998379 | 0.413731808 |
| China | the USA | 1.962014521 | 0.314853905 |
| France | Germany | 0.872980799 | 0.169758916 |
| France | India | 1.298415835 | 0.233196312 |
| France | the USA | 12.1675076 | 0.740251083 |
| Germany | India | 1.359583724 | 0.241528719 |
| Germany | the USA | 1.462861834 | 0.255193867 |
| India | the USA | 12.23811328 | 0.741362071 |
| China | Australia | 21.55692335 | 0.834685035 |
| France | Australia | 9.014194291 | 0.678591268 |
| Germany | Australia | 1.404463415 | 0.247528052 |
| India | Australia | 400.2537462 | 0.989445617 |
| the USA | Australia | 1.610205108 | 0.27385846 |
| France | China | 0.96973082 | 0.185090545 |
| Germany | China | 1.384428847 | 0.244861682 |
| India | China | 4.603678816 | 0.518831241 |
| the USA | China | 2.095148752 | 0.329185646 |
| Germany | France | 1.392759365 | 0.24597267 |
| India | France | 7.267495074 | 0.629930008 |
| the USA | France | 2.039767695 | 0.323297411 |
| India | Germany | 0.536623387 | 0.111654261 |
| the USA | Germany | 0.959749988 | 0.183535163 |
| the USA | India | 2.234095918 | 0.343517387 |

**Table S4** Markov rewards for six countries' inputs and outputs

| In-Out | Markov rewards |
| --- | --- |
| China/In | 4.07 |
| China/Out | 11.32 |
| India/In | 3.97 |
| India/Out | 5.44 |
| France/In | 5.74 |
| France/Out | 4.25 |
| the USA /In | 5.29 |
| the USA /Out | 1.37 |
| Germany/In | 1.18 |
| Germany/Out | 0.20 |
| Australia/In | 8.17 |
| Australia/Out | 5.72 |

**Table S5.** Information on the evolutionary lineage of E25 in this study.

| Genotype | Lineage | Number of sequence | Isolated countries | Isolated years | Nucleotide mean distances(%) |
| --- | --- | --- | --- | --- | --- |
| A | A | 1 | the USA | 1957 | NA |
| D | D1 | 1 | Australia | 2007 | NA |
|  | D2 | 2 | Australia | 2008 | 0 |
|  | D3 | 1 | India | 2010 | NA |
| E | E | 5 | Germany,China | 2008~2010 | 2 |
| F | F1 | 6 | China | 2009~2013 | 5.3 |
|  | F2 | 1 | China | 2013 | NA |
|  | F3 | 3 | China | 2013 | 0.1 |
|  | F4 | 1 | China | 2016 | NA |
|  | F5 | 8 | China | 2013~2018 | 3.7 |
|  | F6 | 1 | China | 2005 | NA |
|  | F7 | 1 | China | 2010 | NA |
|  | F8 | 1 | China | 2008 | NA |
|  | F9 | 1 | the USA | 2018 | NA |
| H | H | 1 | China | 2017 | NA |
| G | G1 | 1 | Australia | 2010 | NA |
|  | G2 | 1 | the USA | 2017 | NA |
| I | I | 1 | the USA | 2016 | NA |

**Table S6**. Recombination results obtained by software RDP4 analysis.

| **Lineage** | **Strains** | **Breakpoint** | | **Major parent** | **Minor parent** | **Unknow parent** | **Region of recombination** | **P-value of the methods** | | | | | | |
| --- | --- | --- | --- | --- | --- | --- | --- | --- | --- | --- | --- | --- | --- | --- |
|  |  | **Beginning breakpoint** | **Ending breakpoint** |  |  |  |  | **RDP** | **Geneconv** | **BootScan** | **MaXChi** | **Chimaera** | **SiScan** | **3Seq** |
| **D1** | **MF678296_australia_2007** | **5391** | **7127** | **NA** | **CVB5_OQ791517.1** | **CVB3_JX476169.1** | **3C,3Dpol** | **4.999×10^-8^** | **NA** | **3.384×10^-9^** | **1.400×10^-9^** | **8.238×10^-10^** | **1.139×10^-16^** | **2.221×10^-5^** |
|  |  | **3433** | **3782** | **E30_JX954435.1** | **NA** | **E6_MZ389225.1** | **2A,2B** | **1.231×10^-2^** | **NA** | **NA** | **1.239×10^-4^** | **2.189×10^-4^** | **NA** | **1.297×10^-2^** |
| **D2** | **MF678297_australia_2008** | **3920** | **4894** | **NA** | **E33_ON383146.1** | **EV-B84_JN797614.1** | **2B,2C** | **3.379×10^-11^** | **NA** | **1.853×10^-15^** | **1.032×10^-4^** | **3.610×10^-5^** | **3.534×10^-16^** | **4.922×10^-6^** |
| **D3** | **MH118026_India_2010** | **5664** | **7225** | **E6_MN145871.1** | **CVB3_KR107057.1** | **NA** | **3C,3Dpol** | **6.873×10^-17^** | **NA** | **5.594×10^-22^** | **9.534×10^-10^** | **3.521×10^-4^** | **3.387×10^-19^** | **1.756×10^-8^** |
| **E** | **MF422564_china_2008** | **6117** | **7335** | **NA** | **E4_MF554740.1** | **CVB5_OQ791520.1** | **3Dpol** | **9.618×10^-16^** | **NA** | **1.301×10^-23^** | **1.374×10^-10^** | **5.537×10^-6^** | **5.443×10^-21^** | **2.122×10^-2^** |
|  |  | **4772** | **5240** | **CVB2_EF174468.1** | **NA** | **CVB2_OQ842440.1** | **2C,3A** | **5.910×10^-15^** | **5.825×10^-6^** | **3.071×10^-14^** | **6.810×10^-4^** | **1.284×10^-4^** | **NA** | **2.156×10^-11^** |
| **F1** | **E25_China_HB12-30** | **4214** | **7406** | **E6_KF042342.1** | **CVB3_KY286529.1** | **NA** | **2C,3A,3B,3C,3Dpol** | **2.988×10^-45^** | **4.183×10^-47^** | **1.010×10^-56^** | **4.320×10^-27^** | **2.607×10^-17^** | **2.220×10^-42^** | **1.513×10^-4^** |
| **F2** | **E25_China_HeN13-345** | **3781** | **5909** | **CVB1_JX976769.1** | **B4_JX308222.1** | **NA** | **2B,2C,3A,3B,3C** | **2.059×10^-34^** | **9.954×10^-43^** | **4.126×10^-43^** | **1.109×10^-19^** | **3.964×10^-14^** | **5.453×10^-28^** | **3.425×10^-10^** |
|  |  | **6986** | **7453** | **0** | **CVB5_HQ998851.1** | **E12_MF083154.1** | **3Dpol** | **2.125×10^-4^** | **3.287×10^-2^** | **1.179×10^-6^** | **6.729×10^-3^** | **1.055×10^-3^** | **4.594×10^-8^** | **NA** |
| **F3** | **E25_China_YN13-114** | **4411** | **7445** | **E30_JX976773.1** | **CVB3_KP036481.1** | **NA** | **2C,3A,3B,3C,3Dpol** | **2.202×10^-70^** | **8.806×10^-88^** | **1.130×10^-87^** | **1.598×10^-29^** | **5.099×10^-23^** | **1.373×10^-50^** | **NA** |
| **F4** | **E25_China_HB16-16** | **4421** | **5748** | **E6_KX619440.1** | **EVB80_MH614923.1** | **NA** | **2C,3A,3B,3C** | **3.306×10^-15^** | **NA** | **3.257×10^-22^** | **6.497×10^-11^** | **5.563×10^-9^** | **2.649×10^-14^** | **8.521×10^-8^** |
| **F5** | **E25_China_JX14-70** | **4267** | **6046** | **E6_HM185056.1** | **B3_GU109481.1** | **NA** | **2C,3A,3B,3C,3Dpol** | **6.339×10^-32^** | **2.495×10^-15^** | **2.855×10^-39^** | **3.607×10^-19^** | **8.241×10^-13^** | **6.408×10^-28^** | **6.217×10^-14^** |
|  |  | **6224** | **7467** | **NA** | **E6_MH185056.1** | **CVB3_FJ357838.1** | **3Dpol** | **1.331×10^-6^** | **NA** | **1.150×10^-10^** | **3.029×10^-6^** | **7.500×10^-7^** | **6.780×10^-22^** | **NA** |
| **F6** | **KX774483_china_2005** | **5318** | **7472** | **NA** | **E30_DQ246620.1** | **CVB4_MW015039.1** | **3B,3C,3Dpol** | **7.834×10^-45^** | **1.082×10^-56^** | **5.280×10^-56^** | **6.571×10^-24^** | **3.454×10^-20^** | **3.734×10^-49^** | **3.147×10^-4^** |
|  |  | **4180** | **5314** | **NA** | **E30_AY948442.1** | **E9_AF524867.1** | **2C,3A** | **5.502×10^-16^** | **1.142×10^-14^** | **1.949×10^-23^** | **3.051×10^-6^** | **1.861×10^-10^** | **3.078×10^-17^** | **NA** |
| **F7** | **JX976772_china_2010** | **4121** | **5262** | **E6_KF042342.1** | **CVB4_MW015040.1** | **NA** | **2C,3A** | **5.935×10^-24^** | **3.033×10^-10^** | **1.996×10^-29^** | **2.469×10^-4^** | **6.477×10^-10^** | **3.204×10^-16^** | **3.637×10^-13^** |
| **F8** | **HM031191_china_2008** | **5810** | **7489** | **NA** | **E6_HM185055.1** | **E11_MN496161.1** | **3C,3Dpol** | **7.650×10^-22^** | **2.702×10^-5^** | **2.449×10^-27^** | **2.468×10^-14^** | **2.024×10^-15^** | **9.714×10^-25^** | **1.099×10^-9^** |
|  |  | **4542** | **5653** | **NA** | **CVB5_JX843811.1** | **E4_FJ172447.1** | **2C,3A,3B,3C** | **4.656×10^-7^** | **NA** | **2.416×10^-11^** | **1.366×10^-7^** | **1.077×10^-3^** | **7.526×10^-11^** | **NA** |
| **F9** | **MK800121_USA_2018** | **4216** | **7425** | **NA** | **CVB2_MH484076.1** | **EV-B84_DQ902712.1** | **2C,3A,3B,3C,3Dpol** | **7.295×10^-55^** | **1.705×10^-79^** | **3.012×10^-80^** | **2.177×10^-30^** | **1.104×10^-22^** | **3.031×10^-52^** | **1.776×10=** |
| **H** | **E25_China_YN17-A31** | **5112** | **7273** | **EVB80_MH614923.1** | **E3_MF422569.1** | **NA** | **3A,3B,3C,3Dpol** | **6.752×10^-13^** | **NA** | **7.833×10^-19^** | **2.244×10^-2^** | **1.096×10^-8^** | **4.086×10^-21^** | **2.456×10^-8^** |
| **G1** | **MF678348_australia_2010** | **5584** | **6929** | **E30_MH484074.1** | **E7_MF678316.1** | **NA** | **3C,3Dpol** | **4.138×10^-24^** | **1.587×10^-5^** | **1.412×10^-27^** | **3.297×10^-16^** | **4.302×10^-18^** | **1.032×10^-20^** | **9.059×10^-14^** |
| **G2** | **MK532311_USA_2017** | **3806** | **4434** | **NA** | **E16_OQ842415.1** | **E30_OP207962.1** | **2B,2C** | **7.927×10^-16^** | **8.912×10^-11^** | **1.901×10^-22^** | **4.284×10^-10^** | **2.683×10^-6^** | **2.218×10^-14^** | **3.777×10^-6^** |
|  |  | **4442** | **4677** | **E29_OL955512.1** | **E18_OQ791559.1** | **NA** | **2C** | **2.148×10^-5^** | **NA** | **4.787×10^-8^** | **3.620×10^-2^** | **NA** | **4.042×10^-5^** | **NA** |
| **I** | **MT347976_USA_2016** | **3458** | **4652** | **E11_OQ969176.1** | **E9_LC321988.1** | **NA** | **2A,2B,2C** | **2.019×10^-4^** | **NA** | **4.528×10^-5^** | **3.548×10^-4^** | **1.642×10^-7^** | **6.996×10^-13^** | **4.041×10^-14^** |
|  |  | **5138** | **5578** | **EVB84_DQ902712.1** | **E11_OQ969176.1** | **NA** | **3A,3B,3C** | **2.104×10^-3^** | **NA** | **1.341×10^-3^** | **4.090×10^-6^** | **3.952×10^-4^** | **7.203×10^-7^** | **NA** |
